# Development of a novel NS1 competitive enzyme-linked immunosorbent assay for the early detection of Zika virus infection

**DOI:** 10.1371/journal.pone.0256220

**Published:** 2021-08-17

**Authors:** Julieta S. Roldán, Alejandro Cassola, Daniela S. Castillo

**Affiliations:** Instituto de Investigaciones Biotecnológicas "Dr. Rodolfo A. Ugalde" (IIBIO), Universidad Nacional de San Martín (UNSAM), Consejo Nacional de Investigaciones Científicas y Técnicas (CONICET), San Martín, Buenos Aires, Argentina; Waseda University: Waseda Daigaku, JAPAN

## Abstract

Zika virus (ZIKV) is a flavivirus that has emerged as a global health threat after the 2015 outbreak in the Americas, where devastating congenital defects were documented. There are currently no vaccines to prevent ZIKV infections nor commercially available clinical diagnostic tests demonstrated to identify ZIKV without cross-reactive interference of related flaviviruses. Early diagnosis is critical when treating symptomatic patients and in preventing ZIKV transmission. In this context, the development of sensitive and accurate diagnostic methods are urgently needed for the detection of ZIKV acute infection. The aim of this study consisted of obtaining monoclonal antibodies (mAbs) against denatured monomeric ZIKV Nonstructural protein 1 (ZNS1), a useful diagnostic marker for flavivirus early detection, in order to develop a highly specific and sensitive ZNS1 indirect competitive ELISA (icELISA). The production of hybridomas secreting ZNS1 mAbs was carried out through immunizations with denatured monomeric ZNS1. We selected 1F5 and 6E2 hybridoma clones, which recognized the heat-denatured ZNS1 hexameric form by indirect ELISA. Cross-reaction studies indicated that these mAbs specifically bind to a ZNS1 linear epitope, and that they do not cross-react with the NS1 protein from other related flaviviruses. The 1F5 mAb enabled the development of a sensitive and reproducible icELISA to detect and quantify small amounts of ZNS1 disease marker in heat-denatured human sera. Here, we establish a reliable 1F5 based-icELISA that constitutes a promising diagnostic tool for control strategies and the prevention of ZIKV propagation.

## Introduction

Zika virus (ZIKV) is a mosquito-borne flavivirus that has gained global attention in the 2015 outbreak in Brazil for its causal link to congenital neurodevelopmental defects, as well as to the Guillain-Barré syndrome in adults [1–4]. ZIKV is primarily spread to humans through the bite of infected *Aedes aegypti* or *Aedes albopictus* mosquitoes [5, 6]. Some reports have also suggested sexual [7, 8] and materno-fetal transmission routes [9]. Disease associated with ZIKV infection varies from asymptomatic cases to mild and non-specific symptoms, which develop 6 to 11 days after infection [10]. These include headache, retro-orbital pain, maculopapular rash, fever, arthralgia, conjunctivitis, edema and vomiting [11]. The virus limited detection window period −up to 10 days after infection− [12], and the current lack of vaccine and effective treatments, emphasize the urgency of the development of sensitive and accurate diagnostic methods for the detection of ZIKV acute infection. Early diagnosis is crucial in preventing ZIKV propagation and can save valuable time for symptomatic patients when receiving treatment [13].

ZIKV belongs to the *Flaviviridae* family, which encloses other major global pathogens such as dengue virus (DENV), West Nile virus (WNV) and yellow fever virus (YFV). The glycoprotein Nonstructural protein 1 (NS1) is highly conserved among flaviviruses. Depending on its glycosylation status, NS1 exists in multiple oligomeric forms and is found at different cellular locations. It occurs as an intracellular membrane associated dimer in infected cells, or as a soluble secreted hexameric lipoparticle [14–18]. NS1 dimer has been involved with early steps of viral replication, whereas hexameric NS1 has been associated with immune evasion [15, 19–21]. High levels of the extracellular NS1 hexamer circulate in the bloodstream of infected patients during the acute phase up to the ninth day after the onset of the symptoms, turning it into the preferred diagnostic marker for flavivirus early detection [13, 22–24]. Therefore, the availability of commercial kits for the detection of NS1 in acute serum provides an alternative to the existing methods such as PCR or serology, which are time-consuming or not appropriate for early diagnosis, respectively [25]. The enzyme-linked immunosorbent assay (ELISA) is a commonly used diagnostic tool due to its relative ease of use, high precission, sensitivity and potential for standarization. Several ELISAs have been developed for the detection of NS1 proteins from DENV, WNV and YFV [26–29], yet there is currently a lack of a ZIKV diagnostic system available in the market for the clinical detection of ZIKV NS1 (ZNS1).

The most important properties of a diagnostic method are their specificity and sensitivity. Developing specific NS1 ELISAs constitutes a challenge due to the NS1 high degree of homology among flaviviruses. For this reason, we aimed at obtaining monoclonal antibodies (mAbs) that would specifically recognize ZNS1 but would not cross-react with the NS1 protein from other related flaviviruses. To address the sensitivity issue, we took advantage from the NS1 quaternary structure. Recent studies have demonstrated that heat dissociation of the NS1 homo-hexamer contained in samples from acute DENV patients is a useful alternative to enhance the sensitivity of commercial ELISA kits by an increase in available monomeric forms of the antigen [30, 31]. With this approach in mind, we have developed and characterized two mAbs −1F5 and 6E2− that specifically recognize ZNS1 in its denatured monomeric conformation. We established a sensitive, reliable and reproducible indirect competitive ELISA (icELISA) using 1F5 mAb. The 1F5-based icELISA enables the detection of small amounts of ZNS1 in heat-denatured supernatant from ZIKV infected cells, as well as in heat-treated ZNS1-spiked human normal serum, and constitutes a promising analytical method for the early detection of ZIKV infection.

## Materials and methods

### Production and purification of recombinant NS1 proteins

Recombinant *Escherichia coli* expressed-NS1 proteins were obtained as 6xHis (His) fusions in pET-22b by subcloning the NS1 open reading frame from pUC57-NS1 constructs (Genscript) into the NdeI and EcoRI sites of the pET-22b vector, generating a His-tagged fusion. The NS1 sequences are listed in S1 Table. Constructs for recombinant protein expression and purification were transformed in *E*. *coli* strain BL21. Cultures transformed with pET22b-NS1 were induced with 1 mM isopropyl β-D-1-thiogalactopyranoside (IPTG) for 3 h at 37° C. Bacterial cell pellets containing NS1 recombinant proteins were resuspended in lysis buffer (50 mM Tris-HCl pH 8.8, 100 mM NaCl, 0.5% Triton X-100). After sonication and centrifugation at 13000 rpm for 10 minutes at 4°C, pellets were washed with lysis buffer, followed by an additional washing step with washing buffer (50 mM Tris-HCl pH 8.8, 100 mM NaCl, 1 M Urea, 1% sucrose). Insoluble NS1 proteins from the pellets were solubilized for 1 h at room temperature (RT) in solubilization buffer (50 mM Tris-HCl pH 8.8, 100 mM NaCl, 8 M Urea, 10 mM 2-mercaptoethanol, 20 mM Imidazole, 50 μg/ml DNase I) and purified by immobilized-metal affinity chromatography (IMAC) using solubilization buffer. After elution with elution buffer (50 mM Tris-HCl pH 8.8, 100 mM NaCl, 8 M Urea, 10 mM 2-mercaptoethanol, 250 mM Imidazole), the most concentrated fractions of NS1 denatured recombinant proteins were pooled and dialyzed against PBS prior to any usage.

HEK293-expressed ZNS1-His recombinant protein was obtained as described previously [32]. When indicated, hexameric ZNS1-His was denatured at 60°C for 15 minutes in the presence of 0.05 M dithiothreitol (DTT). This condition was also applied for denaturation of supernatants from A549 (ATCC CCL-185) infected cells. The denaturation of spiked-serum samples was carried out at 60°C for 15 minutes in the presence of 0.05 M DTT and 2.5% Sodium Dodecyl Sulfate (SDS).

### Immunizations and hybridoma generation

8- to 9-week-old male BALB/c mice were immunized intraperitoneally with 20 μg of denatured *E*.*coli*-expressed ZNS1 emulsified in complete Freund’s adjuvant. Booster injections of 10 μg of denatured *E*.*coli*-expressed ZNS1 in incomplete Freund’s adjuvant were applied 21 and 42 days after the first immunization. Based on the humoral response of a test bleed performed 7 days after the final immunization, the highest BALB/c responder −according to an indirect-enzyme-linked immunosorbent assay (iELISA) (see *indirect-enzyme-linked immunosorbent assay*)− was selected as donor of splenocytes for hybridoma production. Hybridomas were generated by fusion of spleen cells with Sp2/0-Ag14 (ATCC CRL-1581) myeloma cells as described previously [33]. Screening of positive secreting hybridomas was carried out by iELISAs and the selected hybridomas were cloned twice.

### Monoclonal antibodies purification

Monoclonal antibodies were purified by protein G affinity chromatography (mAbia Labs, Argentina) from hybridoma culture supernatants.

### Indirect enzyme-linked immunosorbent assay

Microtiter plates (Nunc Maxisorp 96-well ELISA plates) were coated with 100 μl of denatured *E*.*coli*-expressed ZNS1 (500 ng/well) for hybridoma screening, or with 100 μl of the indicated concentration of denatured *E*.*coli*-expressed NS1 proteins, or native or denatured HEK293-expressed ZNS1 in coating buffer (0.1 M Na_2_HPO_4_ buffer pH 9.5) for 18 h at 4°C. Following incubation in blocking buffer (5% skimmed milk in TBS) for 1 h at 37°C, the plates were further incubated with hybridoma supernatants for screening, or with 0.5 μg/ml of the indicated purified mAb for 1 h at RT in blocking buffer. Following four washing steps in TBS Tween-20 0.05%, plates were further incubated for 1 h at RT with HRP goat anti-mouse IgG Fcγ fragment specific secondary antibody (Jackson Immunoresearch) at a 1:6000 dilution. Finally, plates were washed four times in TBS Tween-20 0.05% and after incubation with the substrate [0.3% H_2_O_2_, 0.1% 3,3’,5,5’-tetramethylbemzidine (TMB) in 0.1 M citric acid pH 5] for 10 to 30 minutes at RT, the reaction was stopped with 0.2 M H_2_SO_4_. The absorbance at 450 nm was measured with a FilterMax F5 Multi-Mode microplate reader (Molecular Devices).

### Isotyping of immunoglobulins

The isotypes of the mAbs were determined with the Mouse Ig Isotyping Ready-SET-Go kit (Affymetrix, eBioscience) according to the manufacturer’s instructions.

### Western blotting

*E*.*coli*-expressed NS1 protein extracts were resolved on 10% SDS-PAGE. After transfer to a nitrocellulose membrane (Hybond-ECL, GE Healthcare), analysis by immunoblotting was performed using 0.2 μg/ml 1F5 or 6E2 purified monoclonal antibodies in blocking buffer (1% skimmed milk in TBS). Bound antibodies were recognized with an Alexa Fluor 680 goat anti-mouse IgG secondary antibody (Invitrogen) at a 1:20000 dilution in blocking buffer. The signal was visualized with an Odyssey Infrared Imager (Li-Cor).

### Indirect immunofluorescence

A549 cells were grown in coverslips and infected with ZIKV strain PRVABC59 (GenBank: KU501215.1), Dengue virus type 1 clone WestPac (GenBank: U88535.1), Dengue virus type 2 strain 16681 (GenBank: M84727.1), Dengue type 3 virus (GenBank: M93130.1), or Dengue virus type 4 Thailand 1978 (GenBank: U18441.1), at a multiplicity of infection (m.o.i.) of 1. After 1 h at 37°C, the inoculum was removed, cells were rinsed twice with PBS and incubated with Modified Eagle Medium (MEM, Life Technologies) supplemented with 1.5% fetal bovine serum (FBS, Natocor) and 50 μg/ml gentamicin sulfate (Sigma-Aldrich) in a 5% CO_2_ humidified atmosphere at 37°C. After 48 h, cells were fixed with 4% paraformaldehyde for 10 minutes at RT, permeabilized with 0.2% Triton X-100 and incubated with 2% bovine serum albumin (BSA) in PBS for 1 h at 37°C. Immunostaining was performed using 1 μg/ml 1F5 or 5 μg/ml 6E2 purified monoclonal antibodies, 1:2000 Alexa Fluor 488 goat anti-mouse IgG secondary antibody (Invitrogen) and 1.5 μg/μl DAPI to visualize nuclei. At least ten fields of each condition were randomly selected for analysis. Image acquisition was performed with a Nikon Eclipse E600 microscope at a magnification of 1000X. Images were processed with the NIH Image J software.

### Stock virus growth

ZIKV stocks were a kind gift from Mayra Alejandra Castañeda (IQUIBICEN-UBA, CONICET), and DENV1-4 stocks were generously provided by Diego Alvarez (IIBIO-UNSAM, CONICET). Briefly, A549 cells were grown in MEM (Life Technologies) supplemented with 10% FBS (Natocor) and 50 μg/ml gentamicin sulfate (Sigma-Aldrich) in a 5% CO_2_ humidified atmosphere. When indicated, cells were infected with ZIKV or DENV1-4 strains as specified in *indirect immunofluorescence*. Supernatants were collected 7 days post-infection (d.p.i.) to harvest virus. Virus was inactivated by UV radiation as described previously [34].

### Competitive indirect enzyme-linked immunosorbent assay

Microtiter plates (Nunc Maxisorp 96-well ELISA plates) were coated with 100 μl of denatured *E*.*coli*-expressed ZNS1 (500 ng/well) in coating buffer (0.1 M Na_2_HPO_4_ buffer pH 9.5) for 18 h at 4°C. Plates were incubated in blocking buffer (5% skimmed milk in TBS) for 1 h at 37°C. Denatured HEK293-expressed ZNS1 −serially diluted in TBS−, denatured supernatants from A549 infected cells, or denatured ZNS1-spiked normal human serum (50 μl), and 50 μl of 2 μg/ml 1F5 mAb in blocking buffer, were preincubated for 1 h at RT and then added to the wells. Detection of antibodies with secondary antibody, reaction development for 15 minutes at RT and absorbance measurement were carried out as described in *indirect-enzyme-linked immunosorbent assay*.

### Statistical analysis

The software GraphPad Prism 5.0 (GraphPad Software, La Jolla, CA, USA) was used for the non linear fitting of the standard curves to a 4 parameter logistic regression and for the calculation of the IC50 parameter. Percentage of maximum response was calculated as (OD 450 nm/maximum OD 450 nm of the run)*100. Percentage of inhibition was determined, assuming the OD 450 nm reading in the non-inhibited well as 100% of binding, according to the following formula: % inhibition = 100 − [(OD 450 nm inhibited well/OD 450 nm non-inhibited well)*100]. The CR (cross-reactivity) values were calculated as (IC50 of the antigen for which the mAb was developed/IC50 of the antigen analyzed)*100. The limit of detection (LOD) and the limit of quantification (LOQ) were calculated by the following formulas: LOD = mean blank samples±3*SD blank samples and LOQ = mean blank samples±10*SD blank samples [35]. Repeatability (intra-plate variability) was assessed by measuring the standard curve six times for the same denatured HEK293-expressed ZNS1 sample on the same ELISA plate. Reproducibility (inter-plate variability) was calculated by measuring the standard curve for different denatured HEK293-expressed ZNS1 samples on two different ELISA plates on different days. The CV (coefficient of variation) was estimated as follows: standard deviation/mean*100.

### Ethical statement

The protocol of animal immunization followed in this study was approved by the Committee on the Ethics of Animal Experiments of the Universidad Nacional de San Martín (Resolution No. 03/2017), according to the recommendations of the Guide for the Care and Use of Laboratory Animals of the National Institutes of Health.

All the human sera analyzed in this study came from a panel of 30 serum samples obtained from healthy individuals provided by the Instituto Nacional de Parasitología "Dr. Mario Fatala Chaben". The serum samples had been codified upon collection in order to ensure anonymity of the patients.

## Results

### Production and characterization of anti-ZNS1 monoclonal antibodies

With the aim to obtain hybridomas secreting specific antibodies against ZNS1, mice were immunized intraperitoneally with denatured monomeric *E*.*coli*-expressed ZNS1. Hybridomas were generated by fusion of spleen cells from the immunized mice and myeloma cells. In order to select hybridoma clones that would recognize the denatured monomeric conformation, screening by indirect ELISA (iELISA) was performed with the same antigen used for immunizations. The hybridomas 1F5 and 6E2, which expressed high affinity mAbs against ZNS1, were selected and cloned twice. We determined that the isotypes of the mAbs were IgG2a for 1F5 and IgG1 for 6E2, both of them containing kappa light chains (Table 1).

**Table 1:** Isotypes of 1F5 and 6E2 mAbs.

	IgG1	IgG2a	IgG2b	IgG3	IgA	IgM	Kappa	Lambda
**1F5**	0.08	**2.69**	0.07	0.09	0.07	0.10	**1.99**	0.08
**6E2**	**2.78**	0.14	0.07	0.09	0.00	0.12	**2.11**	0.13

**Fig 1:**
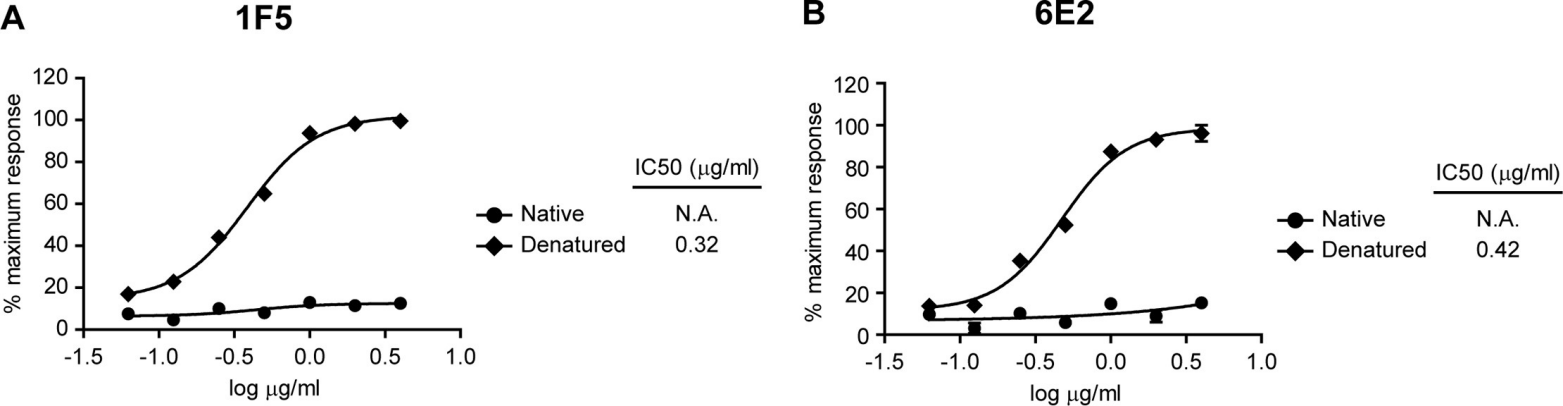
Relative affinity of 1F5 and 6E2 mAbs for the denatured monomeric and the native hexameric ZNS1 conformations. Standard curves of denatured monomeric or native hexameric HEK293-expressed ZNS1 detected by iELISA with **(A)** 1F5 or **(B)** 6E2 mAbs. Each point of the curve represents mean±SEM of three sample replicates. IC50 values of the mAbs are indicated.

Given that the developed mAbs were selected with denatured monomeric *E*.*coli*-expressed ZNS1, we wanted to confirm that they were also capable of recognizing denatured ZNS1 monomer with its corresponding post-translational modifications, as it would be found in a ZIKV infected patient’s serum subjected to denaturation. For this purpose, we studied 1F5 and 6E2 relative sensitivity for denatured monomeric and native hexameric HEK293-expressed ZNS1. Optimal NS1 denaturation conditions were established considering that this antigen would be detected from ZIKV infected patients sera. It is known that serum heated at 100°C forms a clot or gel (S1A Fig). For that reason, ZNS1 denaturation was carried out at a lower temperature (60°C) for 15 minutes, a condition that hinders the formation of a coagulum (S1B Fig). The reductant agent dithiothreitol (DTT) at a 0.05 M concentration was added to help in the denaturation of the ZNS1 hexameric recombinant protein (S1E and S1F Fig). Therefore, to test the relative affinity of 1F5 and 6E2 mAbs for native or denatured ZNS1 obtained from the mammalian expression system, different concentrations of both antigens were immobilized and detected with the mAbs by iELISA (Fig 1). The relative affinity of each mAb for the antigen was quantified by calculation of the concentration of the antigen conferring a 50% reduction of the peak signal in the ELISA (IC50). 1F5 exhibited a slightly higher affinity against denatured HEK293-expressed ZNS1 than 6E2, and none of them showed reactivity towards the hexameric HEK293-expressed ZNS1 antigen. These results indicate that 1F5 and 6E2 mAbs bind to exposed linear epitopes in denatured monomeric ZNS1, which are fully unavailable for mAb recognition in the ZNS1 hexamer.

### Specificity of 1F5 and 6E2 monoclonal antibodies

**Fig 2:**
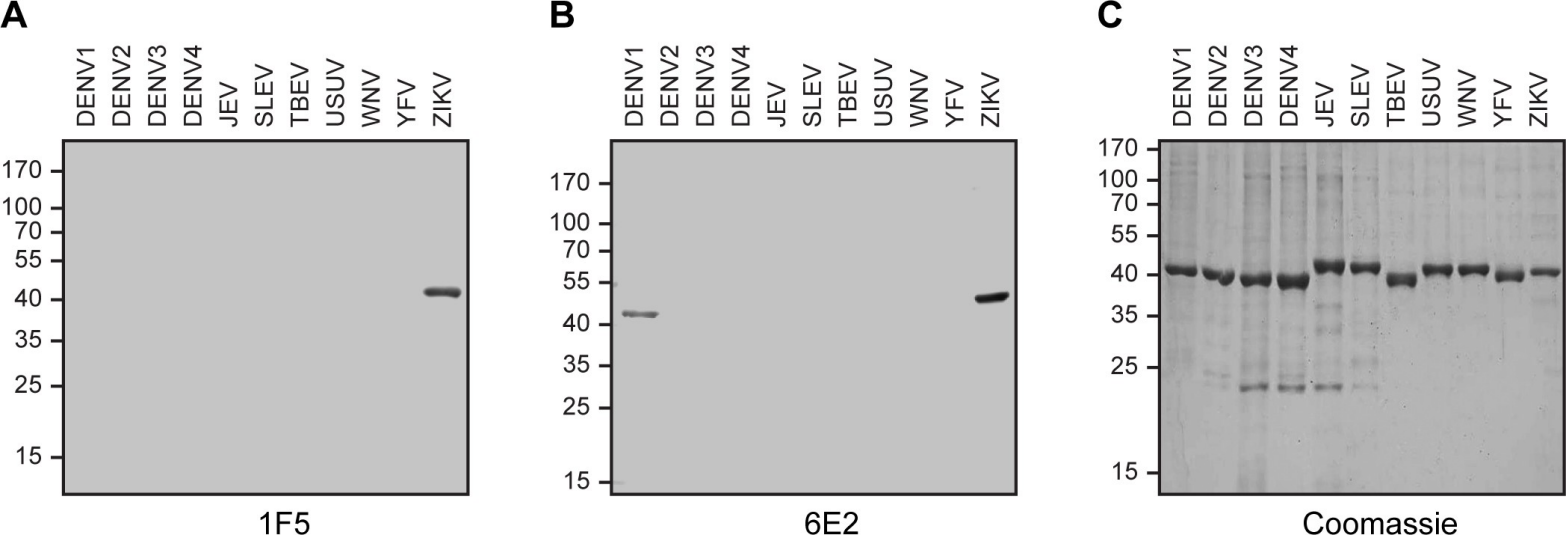
Western blot analysis of flavivirus recombinant NS1 proteins with 1F5 and 6E2 mAbs. SDS-PAGE analysis of *E*.*coli*-expressed DENV1-4, JEV, SLEV, TBEV, USUV, WNV, YFV and ZIKV recombinant NS1 protein samples analyzed by immunoblot using **(A)** 1F5 or **(B)** 6E2 mAbs, or by **(C)** Coomassie brilliant blue staining. The position of the molecular mass standards is indicated on the left.

**Fig 3:**
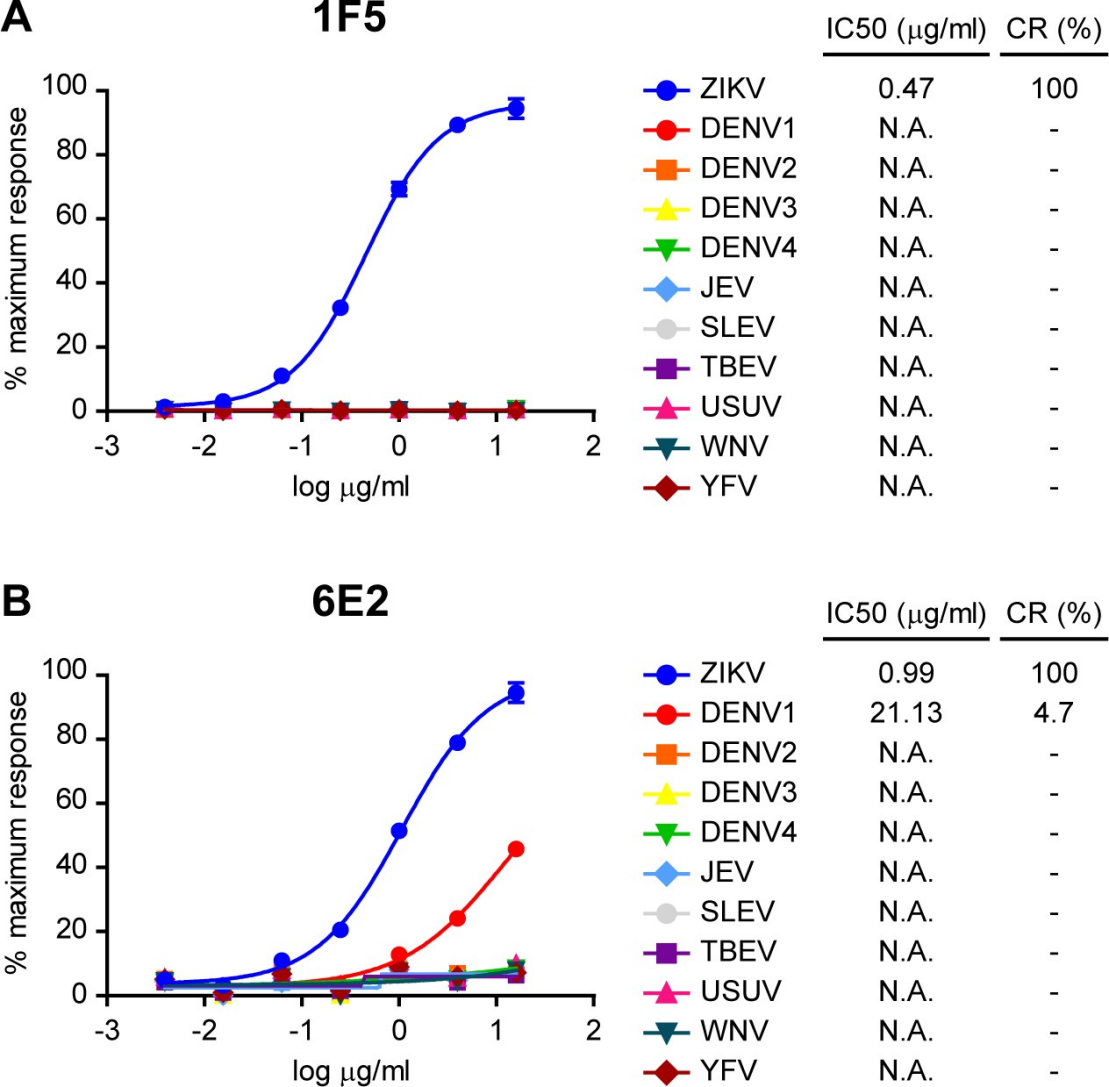
Comparative reactivity of flavivirus recombinant NS1 proteins by indirect ELISA with 1F5 and 6E2 mAbs. Standard curves of denatured *E*.*coli*-expressed DENV1-4, JEV, SLEV, TBEV, USUV, WNV, YFV and ZIKV recombinant NS1 proteins by iELISA with **(A)** 1F5 or **(B)** 6E2 mAbs. Each point of the curve represents mean±SEM of three sample replicates. IC50 and CR values of the mAbs are indicated. N.A.: not applicable.

**Fig 4:**
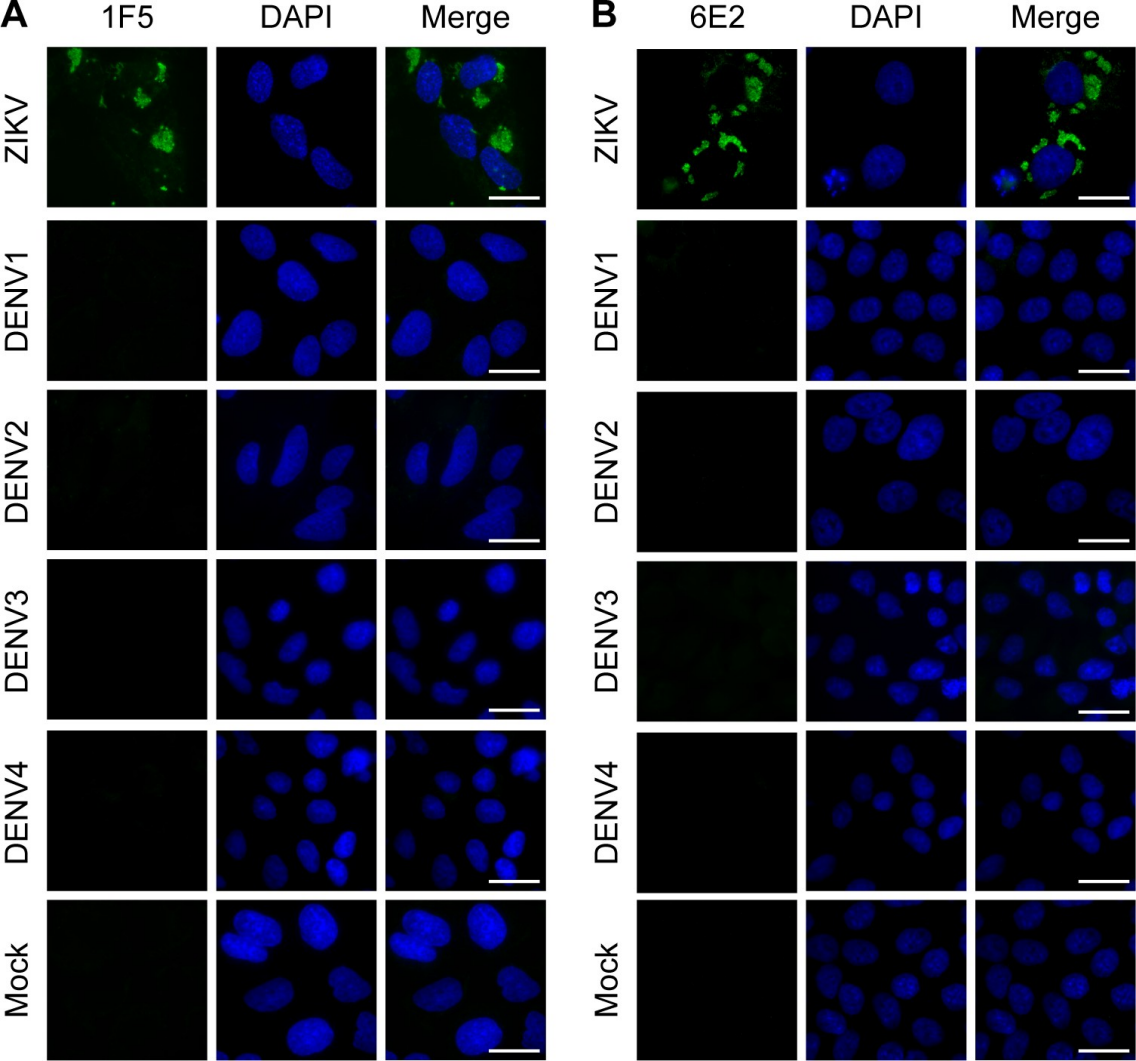
Cross-reactivity analysis of 1F5 and 6E2 mAbs against different flaviviruses in infected cells. Immunostaining of ZIKV and DENV1-4 A549 infected cells with **(A)** 1F5 or **(B)** 6E2 mAbs. Uninfected A549 cells (mock) were used as the negative control. Scale bar, 25 μm.

To evaluate the specificity of the developed mAbs, we first assessed their reactivity by Immunoblot analysis against *E*.*coli* expressed-NS1 proteins from DENV1-4, Japanese encephalitis virus (JEV), Saint Louis encephalitis virus (SLEV), tick-borne encephalitis virus (TBEV), Usutu virus (USUV), WNV and YFV related flaviviruses (Fig 2). Western blot assays showed that 1F5 specifically detected ZNS1 and did not recognize the NS1 protein of the other flaviviruses analyzed (Fig 2A). On the other hand, 6E2 presented some crossreactivity with NS1 from DENV1 as well as with ZNS1 as expected (Fig 2B). To further gather quantitative data about the capacity of 1F5 and 6E2 mAbs to detect NS1 from different flaviviruses, we carried out an iELISA with the NS1 proteins assayed previously (Fig 3). Both mAbs detected denatured monomeric *E*.*coli* expressed-ZNS1 with a high sensitivity, which was twice higher for 1F5 (Fig 3A). Besides, 6E2 mAb also showed some crossreactivity (4.7%) with DENV1 NS1 protein (Fig 3B), consistent with the Western blot results (Fig 2B). To finally characterize 1F5 and 6E2 mAbs, we tested their reactivity against ZIKV and DENV1-4 A549 infected cells by indirect immunofluoresence (Fig 4). A granular staining at the perinuclear location typical of the NS1 protein was observed for ZIKV infected cells for both mAbs, which was not detected in DENV1-4 infected cells. It is known that the predominant intracellular NS1 conformation of flavivirus infected cells is the NS1 dimer [16]. Therefore, these results suggest that 1F5 and 6E2 recognize a linear epitope that is exposed in the ZNS1 dimer. 6E2 lack of crossreactivity with NS1 from DENV1 A549 infected cells might be due to the low sensitivity of the immunostaining technique or to the epitope unaccessibility in the dimeric conformation. Taken together, these results indicate that 1F5 and 6E2 mAbs are highly specific to a ZNS1 linear epitope and, with the exception of DENV1 for 6E2, do not present cross-reactions with the NS1 protein of other related flaviviruses that are associated with similar clinical manifestations.

### Development of an indirect competitive enzyme-linked immunosorbent assay using 1F5 monoclonal antibody

**Fig 5:**
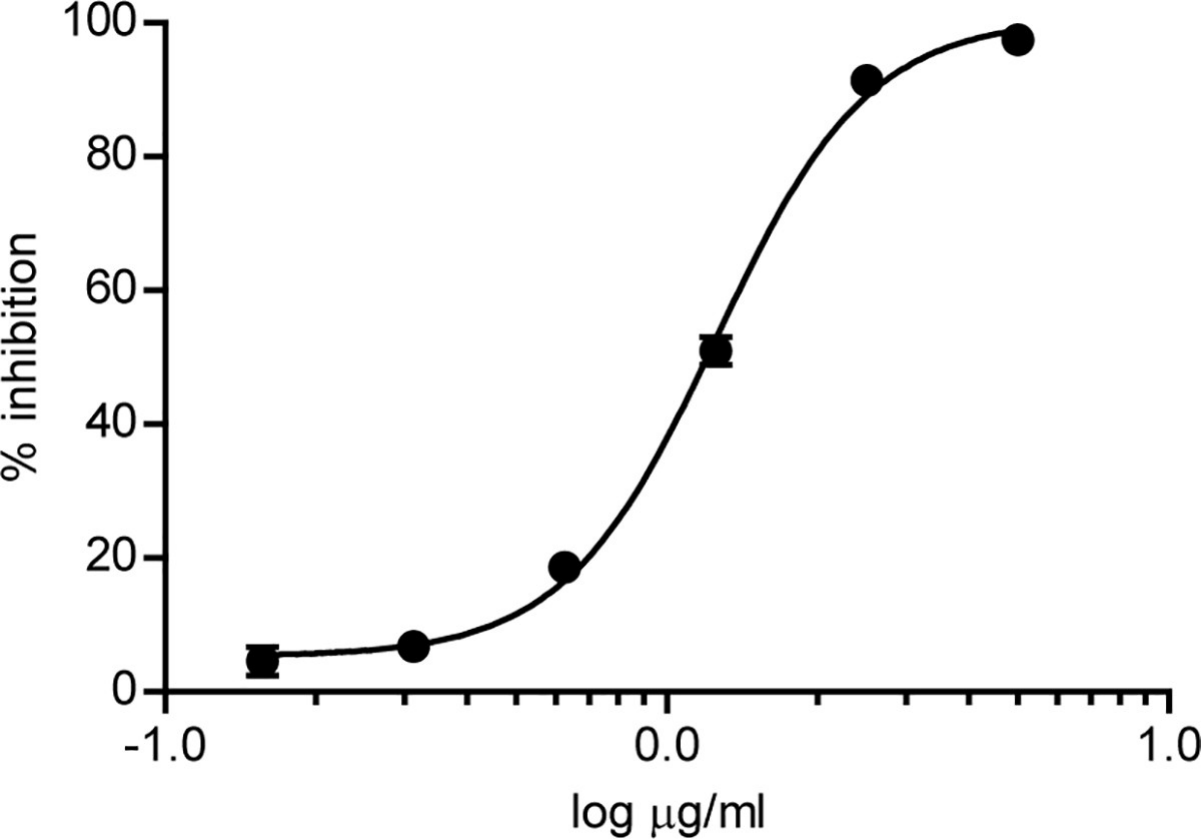
Standard curve for the detection of ZNS1 by indirect competitive ELISA with 1F5 mAb. Standard curve of the icELISA to detect denatured ZNS1 using 1F5 mAb, calculated with a 4 parameter logistic regression fitting with the following equation: Y = Bottom + (Top-Bottom)/(1+10^((LogEC50-X)*Hill slope)), where Bottom = 5.28, Top = 100.6, EC50 = 1.26 and Hill slope = 2.87. Each point of the curve represents mean±SEM of six sample replicates.

Previous studies have described an optimization approach of the sensitivity of DENV NS1 commercial ELISAs by subjecting sera from acute patients to heat treatment, in order to increase the antigen available monomeric forms [30, 31]. We decided to carry out a similar strategy to develop a ZNS1 icELISA, utilizing 1F5 mAb for its higher affinity and specificity towards the diagnostic marker. In our icELISA the monomeric denatured antigen fixed on the plate and the soluble denatured antigen in the sample compete for binding to the anti-ZNS1 1F5 mAb, which is subsequently detected with a HRP-secondary antibody. Thus, a decrease in the signal indicates the presence of the antigen in the sample analyzed. We performed several icELISAs to establish the optimal assay conditions. A denatured monomeric *E*.*coli* expressed-ZNS1 concentration of 5 μg/ml immobilized on the plate and a preincubation of 1 μg/ml 1F5 mAb with the denatured sample for 1 h at room temperature were found to be optimum. Denatured HEK293-expressed ZNS1 was used as the internal standard to develop the ZNS1 icELISA. We determined a good correlation to the data (R^2^ = 0.99) for the 1F5-icELISA standard curve built under the stated conditions, which ranged from 0.156 μg/ml to 5 μg/ml (S2 Fig). Standard ZNS1 concentrations below 0.156 μg/ml in the calibration curve showed a low dose ’hook’ effect, probably due to the formation of 1F5-ZNS1 multimeric complexes [36]. The denatured ZNS1 concentration that inhibited 50% total binding of the mAb (IC50) in the standard curve of the icELISA was 1.20 μg/ml (Fig 5). The resulting 1F5-based icELISA had a limit of detection (LOD) of 0.34 μg/ml and a limit of quantification (LOQ) of 0.70 μg/ml (S3 Fig). The repeatability and reproducibility of the method were estimated by calculating the intraassay and interassay variation from several standard curves carried out on the same or on different ELISA plates, respectively (Table 2). For the denatured ZNS1 standards between 0.625 and 5 μg/ml, we determined an intraassay CV of 0.55–14.19% and an interassay CV of 0.19–7.04%. These results indicate that our 1F5-based ZNS1 icELISA meet the requirements of intraassay and interassay CV used for the validation of bioanalytical methods [37].

**Table 2:** Intraassay and interassay variation of denatured ZNS1 standards analyzed by indirect competitive ELISA with 1F5 mAb.

	Intraassay (n = 6)		Interassay (n = 2)	
ZNS1 (μg/ml)	Inhibition (%)^1^	CV (%)^2^	Inhibition (%)^1^	CV (%)^2^
**5**	97.44±0.54	0.55	97.31±0.18	0.19
**2.5**	91.37±1.43	1.56	89.60±2.49	2.78
**1.25**	50.99±5.12	10.03	49.58±2.00	4.04
**0.625**	18.59±2.64	14.19	17.71±1.25	7.04

^1^mean±SD.

^2^Coefficient of Variation.

To evaluate the suitability of the developed ZNS1 icELISA for the diagnosis of ZIKV acute infection, we first tested heat-denatured supernatants of A549 cells infected with molecularly defined ZIKV or DENV1-4 isolates. As expected, ZNS1 was detected in the supernatant from ZIKV infected cells, but no signal was observed in the supernatant from any DENV infected cells (Table 3). We further validated our icELISA through spike-and-recovery tests. Denaturation of human sera was performed as described previously, at 60°C for 15 minutes in the presence of DTT, yet with the addition of the surfactant agent Sodium Dodecyl Sulfate (SDS) at a 2.5% w/v concentration. This ionic detergent is known to bind to proteins through ionic and hydrophobic interactions, helping in their solubilization by altering their secondary and tertiary structure [38]. Since the addition of SDS in human sera improved protein solubilization and prevented the formation of a clot during sera heating at 60° for 15 minutes in the presence of DTT (S1C and S1D Fig), and did not affect the mAb binding affinity towards denatured ZNS1 hexameric recombinant antigen (S1E and S1F Fig), we decided to include it during the human sera denaturation process. Therefore, normal human sera spiked with low (0.75 μg/ml), medium (1 μg/ml) or high (2 μg/ml) levels of native hexameric HEK293-expressed ZNS1 and subjected to denaturation were analyzed by the 1F5-based icELISA (S2 Table). The average recoveries at the three supplemented levels ranged from 82.3 to 116.0% and the CV varied from 2.2 to 15.0% (Table 4). All recoveries lay within the preset tolerance range of 80–120% [39], which was sufficient as a proof of concept regarding its utility in diagnosis. Overall, these data show that the ZNS1 1F5-icELISA is sensitive, reliable and reproducible.

**Table 3:** ZNS1 detection in supernatants from A549 infected cells by indirect competitive ELISA with 1F5 mAb.

Isolate	ZNS1 (μg/ml)
**ZIKV**	<LOQ
**DENV1**	n.d.^1^
**DENV2**	n.d.^1^
**DENV3**	n.d.^1^
**DENV4**	n.d.^1^
**Mock** ^2^	n.d.^1^

^1^n.d.: not detected.

^2^uninfected A549 cells.

**Table 4:** Recovery analysis of normal human sera supplemented with hexameric ZNS1 by 1F5 based-indirect competitive ELISA.

Spike level	Recovery (%)^1^	CV (%)^2^
**Low (0.75 μg/ml)**	116.0±17.4	15.0
**Medium (1 μg/ml)**	101.8±14.3	14.1
**High (2 μg/ml)**	82.3±1.8	2.2

^1^Mean±SD (n = 4).

^2^Coefficient of Variation.

## Discussion

Originally discovered in 1947, ZIKV received little attention until the 2015 outbreak in Brazil, where many devastating severe diseases −such as the Guillain Barré syndrome in adults and congenital malformations in the fetuses of infected pregnant women− were associated with ZIKV infection [1, 40]. The size of the outbreak and the severity of associated birth defects prompted the World Health Organization to declare ZIKV a Public Health Emergency of International Concern in 2016 [41]. Millions of ZIKV cases have now been reported from 86 countries and, as with many other arboviral diseases, the ZIKV epidemic is further aggravated by disproportionate vector risk near low resource populations least equipped to fully detect and handle outbreaks [42, 43]. Given that other flaviviruses may cause similar symptoms and can occur in coincident epidemics, the development of tools for their differential diagnosis and epidemiological monitoring is crucial [43]. A rapid immunochromatographic lateral flow test specific to ZIKV has recently been described by Bosch et al. [44]. Nonetheless, there are currently no commercially available clinical diagnostic tests demonstrated to identify ZIKV without cross-reactive interference of related flaviviruses, nor vaccines to prevent ZIKV infections. NS1, a highly conserved non-structural protein among these viruses, is considered a useful diagnostic marker because of its release as a hexameric form into the bloodstream of infected patients during the acute phase, up to the ninth day after the onset of the symptoms [13, 22–24]. In view of the urgent need of diagnostic tests to detect and distinguish ZIKV from related flaviviruses, we aimed to obtain mAbs against heat-denatured ZNS1 in order to develop a sensitive and accurate icELISA to detect and quantify this biological marker of acute viral infection.

Immunochemical based-analytical methods, specially ELISA, are the most frequently applied for the detection of disease markers in the diagnostic industry. The overall performance of an ELISA-based method for diagnosis relies on two factors: the specificity with which the antibody or antibodies used in the ELISA detect the antigen in the matrix analyzed, and the sensitivity of the assay towards measuring the smallest amount of target analyte under the standard conditions defined [45].

In this study, we have developed and thoroughly characterized 1F5 and 6E2 mAbs, which were designed to recognize ZNS1 in its denatured monomeric conformation. As it was determined by iELISA, they showed reactivity towards denatured HEK293-expressed ZNS1, which was higher for 1F5 mAb. Nevertheless, none of them recognized the hexameric HEK293-expressed ZNS1 form, indicating that DTT and heat treatment expose the epitopes detected by 1F5 and 6E2 mAbs, that are not accessible for mAb recognition in the homo-hexamer antigen. Cross-reaction studies through Western blotting, iELISA and immunofluoresence staining indicated that 1F5 and 6E2 mAbs recognize a ZNS1 linear epitope and, with the exception of DENV1 for 6E2, do not present cross-reactions with the NS1 protein of DENV1-4, JEV, SLEV, TBEV, USUV, WNV and YFV related flaviviruses. Therefore, 1F5 mAb was chosen as a suitable candidate to develop an immunochemical assay to detect ZNS1 for its affinity and specificity against this diagnostic marker.

Previous work by Lima et al. and Buonora et al. described an alternative to optimize the sensitivity of DENV NS1 commercial ELISAs by subjecting sera from acute patients to heat dissociation, which resulted in an increase in available monomeric forms of the antigen [30, 31]. However, in these studies, the sera was diluted 1/3 with RNA/DNAse free water in order to avoid the formation of a clot during heating at 100°C. This dilution step certainly rendered a lower concentration of NS1 protein in the samples. As a proof of concept, and with the aim to enhance the sensitivity of our ZNS1 immunoassay, we sought to carry out a similar approach by establishing a sera denaturing condition by heat treatment that prevented the generation of a clot, yet avoiding the dilution step. We determined that heating at 60°C for 15 minutes in the presence of the reductant reagent DTT and the surfactant SDS as the optimal sera denaturing condition, since it circumvented serum thermal coagulation. Thus, we developed an icELISA based on 1F5 mAb with monomeric *E*.*coli* expressed-ZNS1 fixed on the plate and HEK293-expressed denatured ZNS1 hexamer to build the standard curve. The ELISA system described here showed an IC50 value of 1.20 μg/ml and high repeatability and reproducibility, with intraassay and interassay CV values lower than 20% [37]. We determined that the LOD and LOQ of the 1F5 based-icELISA are 0.34 and 0.70 μg/ml, respectively. Although the ZNS1 sandwich ELISA described by Zhang et al. was not validated regarding its LOD and LOQ parameters, we established an immunoassay with a sensitivity in the same order of magnitude as this group [46]. Sandwich ELISA is the commonly adopted ELISA method for the detection and quantification of disease markers due to its higher sensitivity. Nevertheless, we demonstrated that a ZNS1 heat dissociation strategy led to the development of an icELISA as sensitive as a sandwich ELISA, with the additional advantage of detecting monomeric ZNS1from undiluted sera in the presence of DTT and SDS.

The accuracy of the icELISA based on 1F5 mAb was assessed by the spike-and-recovery tests. The average recoveries were between the ideal range from 80 to 120% [39]. We also validated the 1F5-icELISA measuring the concentration of ZNS1 from supernatants of ZIKV infected cells, confirming the assay’s specificity as it did not detect any NS1 from cells infected with DENV1-4 isolates.

In conclusion, mAbs against ZNS1 in its denatured monomeric conformation were selected and characterized, and enabled the development of an accurate and sensitive immunodetection test for the diagnosis of ZIKV acute infection. The future of ZIKV is unpredictable, yet its recent spread and associated severe diseases suggest that this virus may become a very serious global public health problem. In the absence of a vaccine, early diagnosis is crucial in the treatment of symptomatic patients. The present work establishes a valid icELISA, based on 1F5 mAb, that allows the detection and quantification of small amounts of ZNS1 in human serum, and stands as a promising diagnostic tool for control strategies and the prevention of ZIKV propagation.

## Supporting information

S1 Fig Determination of the optimal sera denaturation condition. **(A-D)** Normal human sera denatured at the described conditions: **(A)** 5 minutes at 100°C, **(B)** 15 minutes at 60°C, **(C)** 15 minutes at 60°C in the presence of 0.05 M DTT, and **(D)** 15 minutes at 60°C in the presence of 0.05 M DTT and 2.5% SDS. C: clot. **(E and F)** Standard curves of HEK293-expressed ZNS1 hexameric protein subjected to different denaturation conditions, detected by iELISA with **(E)** 1F5 or **(F)** 6E2 mAbs. Each point of the curve represents mean±SEM of three sample replicates. IC50 values of the mAbs are indicated.
(TIF)

S2 Fig Determination of the standard curve range of the 1F5 indirect competitive ELISA. Standard curve of the 1F5 based-icELISA to detect denatured ZNS1. The grey area shows ZNS1 concentrations that exhibited a low dose ’hook’ effect. The dotted lines indicate the lowest (0.156 μg/ml) and highest (5 μg/ml) ZNS1 concentrations considered to build the standard curve. Each point of the curve represents mean±SEM of six sample replicates.
(TIF)

S3 Fig Determination of the limits of detection and quantification for the 1F5 indirect competitive ELISA. The limit of detection (LOD) and the limit of quantification (LOQ) for the determination of denatured ZNS1 by icELISA with 1F5 mAb were obtained measuring the concentration of 6 different normal human sera (blank samples) and calculated by the following formulas: LOD = mean blank samples±3*SD blank samples, and LOQ = mean blank samples±10*SD blank samples. The dotted line represents the mean of the blank samples analyzed.
(TIF)

S1 Table Flavivirus NS1 sequences subcloned into the NdeI/EcoRI sites of the pET-22b vector. (DOCX)

S2 Table Recovery assay of normal human sera spiked with hexameric ZNS1 by 1F5 indirect competitive ELISA. (DOCX)

S1 Raw images (TIF)
