# Supplementary figures and images for: Development of a novel NS1 competitive enzyme-linked immunosorbent assay for the early detection of Zika virus infection
Source: PLoS One. 2021 Aug 17;16(8):e0256220. doi: 10.1371/journal.pone.0256220 (PMC8370630; doi:10.1371/journal.pone.0256220)

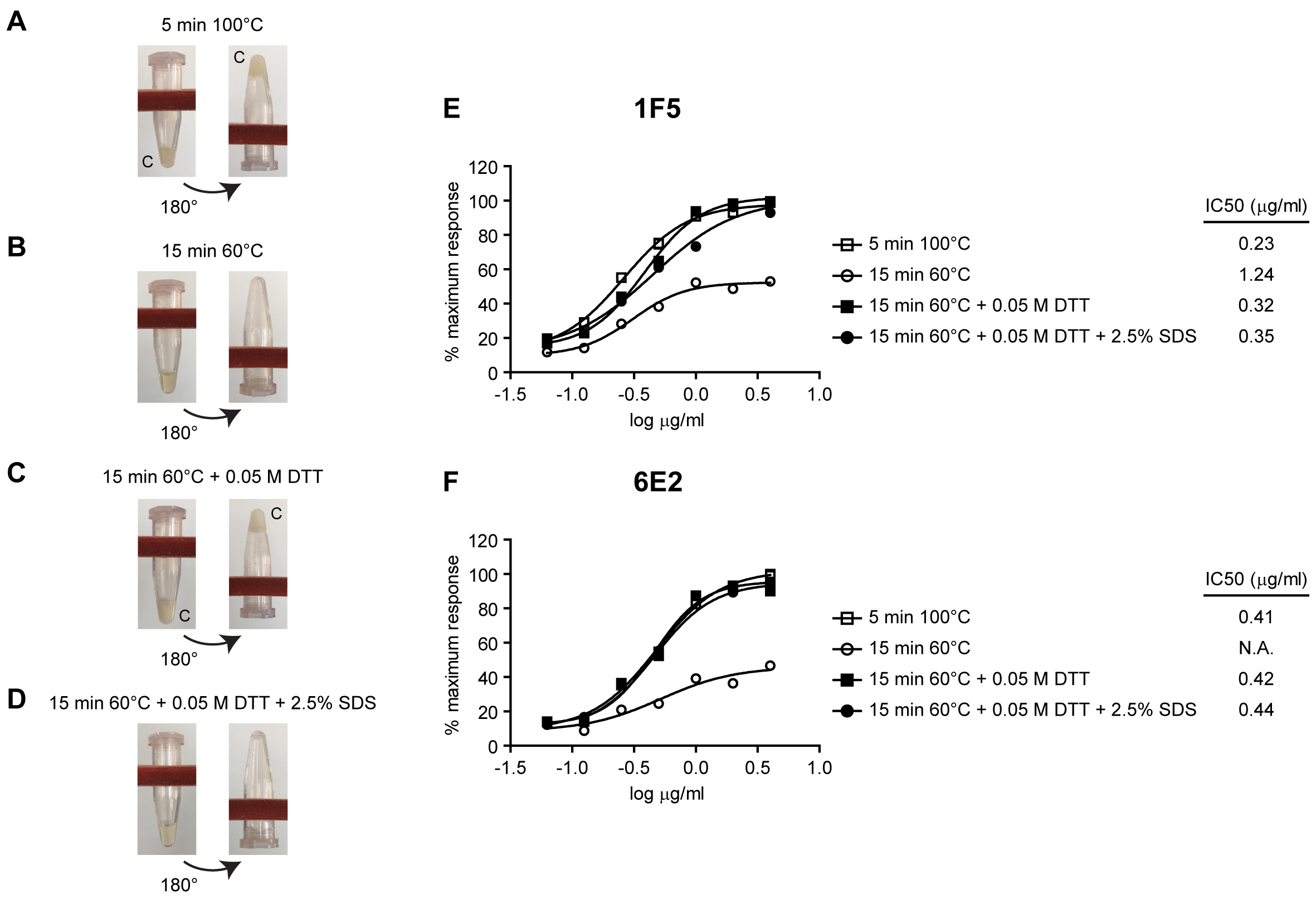

Supplement: S1 Fig — (A-D) Normal human sera denatured at the described conditions: (A) 5 minutes at 100°C, (B) 15 minutes at 60°C, (C) 15 minutes at 60°C in the presence of 0.05 M DTT, and (D) 15 minutes at 60°C in the presence of 0.05 M DTT and 2.5% SDS. C: clot. (E and F) Standard curves of HEK293-expressed ZNS1 hexameric protein subjected to different denaturation conditions, detected by iELISA with (E) 1F5 or (F) 6E2 mAbs. Each point of the curve represents mean±SEM of three sample replicates. IC50 values of the mAbs are indicated. (TIF) [file pone.0256220.s001.tif]

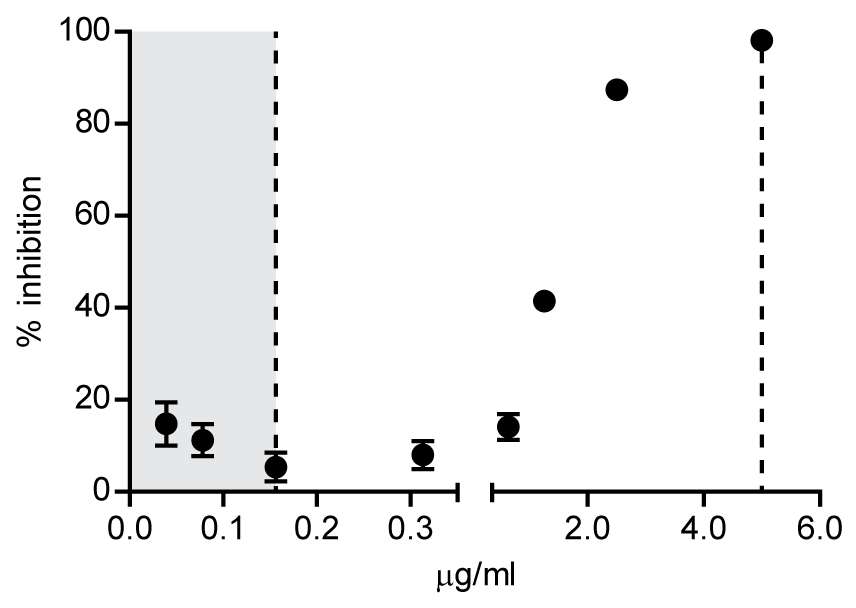

Supplement: S2 Fig — Standard curve of the 1F5 based-icELISA to detect denatured ZNS1. The grey area shows ZNS1 concentrations that exhibited a low dose ’hook’ effect. The dotted lines indicate the lowest (0.156 μg/ml) and highest (5 μg/ml) ZNS1 concentrations considered to build the standard curve. Each point of the curve represents mean±SEM of six sample replicates. (TIF) [file pone.0256220.s002.tif]

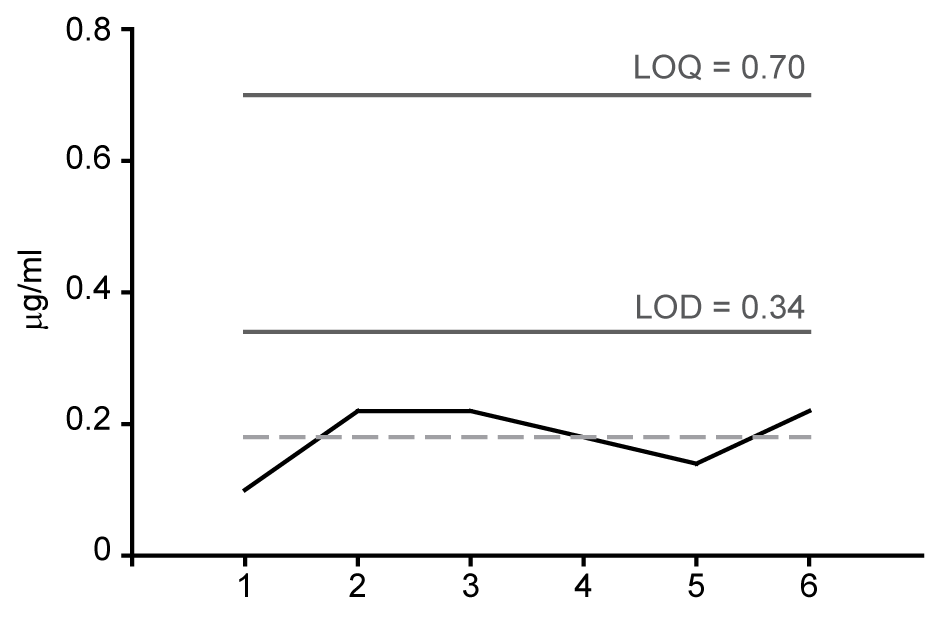

Supplement: S3 Fig — The limit of detection (LOD) and the limit of quantification (LOQ) for the determination of denatured ZNS1 by icELISA with 1F5 mAb were obtained measuring the concentration of 6 different normal human sera (blank samples) and calculated by the following formulas: LOD = mean blank samples±3*SD blank samples, and LOQ = mean blank samples±10*SD blank samples. The dotted line represents the mean of the blank samples analyzed. (TIF) [file pone.0256220.s003.tif]

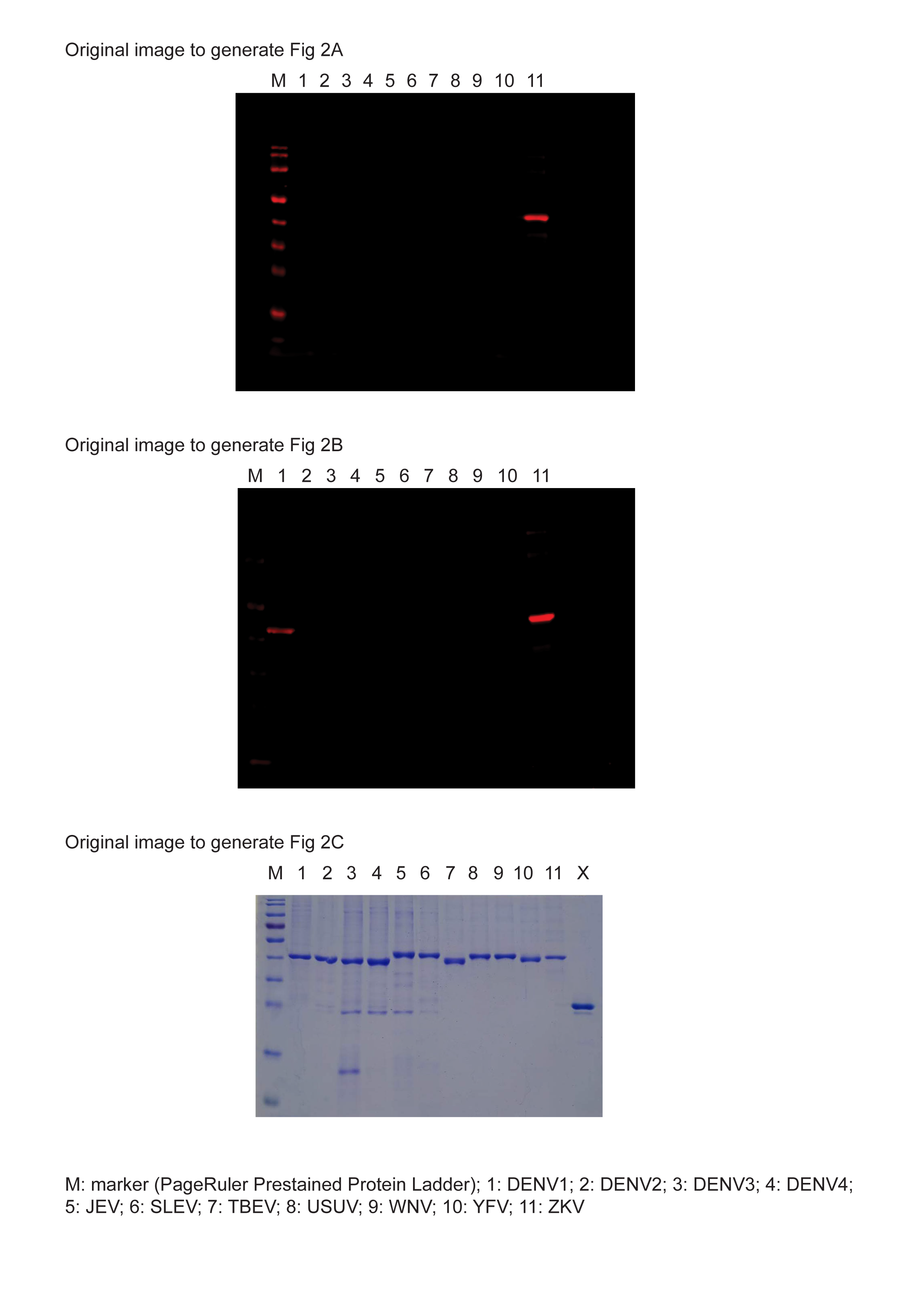

Supplement: S1 Raw images — (TIF) [file pone.0256220.s006.tif]
