# Supplementary material for: Development of a novel NS1 competitive enzyme-linked immunosorbent assay for the early detection of Zika virus infection
Source: PLoS One. 2021 Aug 17;16(8):e0256220. doi: 10.1371/journal.pone.0256220 (PMC8370630; doi:10.1371/journal.pone.0256220)
Supplement: S1 Table — (DOCX) [file pone.0256220.s004.docx]

| **Flavivirus** | **UniProt Entry** | **NS1 position** |
| --- | --- | --- |
| DENV1 | P27909 | 776-1127 |
| DENV2 | P29991 | 776-1127 |
| DENV3 | Q6YMS3 | 774-1125 |
| DENV4 | Q2YHF0 | 775-1126 |
| JEV | P27395 | 795-1146 |
| SLEV | P09732 | 790-1141 |
| TBEV | Q01299 | 777-1128 |
| USUV | Q5WPU5 | 794-1145 |
| WNV | P06935 | 788-1139 |
| YFV | Q6J3P1 | 779-1130 |
| ZIKV | Q32ZE1 | 791-1142 |
