# Supplementary material for: Development of a novel NS1 competitive enzyme-linked immunosorbent assay for the early detection of Zika virus infection
Source: PLoS One. 2021 Aug 17;16(8):e0256220. doi: 10.1371/journal.pone.0256220 (PMC8370630; doi:10.1371/journal.pone.0256220)
Supplement: S2 Table — (DOCX) [file pone.0256220.s005.docx]

| **Spike level** | **Sample**^1^ | **Mean±SD (μg/ml)**^2^ | **CV (%)**^3^ |  | **Recovery (%)** |
| --- | --- | --- | --- | --- | --- |
| **Low (0.75 μg/ml)** | NHS A | 0.719±0.016 | 2.25 |  | 95.9 |
|  | NHS B | 0.803±0.055 | 6.82 |  | 107.3 |
|  | NHS C | 0.996±0.007 | 0.67 |  | 132.7 |
|  | NHS D | 0.961±0.003 | 0.29 |  | 128.2 |
| **Mean Recovery±SD (%)** | | | |  | **116.0±17.4** |
| **Medium (1 μg/ml)** | NHS E | 1.159±0.093 | 7.99 |  | 115.9 |
|  | NHS F | 0.820±0.029 | 3.57 |  | 82.0 |
|  | NHS C | 1.028±0.027 | 2.62 |  | 102.8 |
|  | NHS G | 1.064±0.009 | 0.84 |  | 106.4 |
| **Mean Recovery±SD (%)** | | | |  | **101.8±14.3** |
| **High (2 μg/ml)** | NHS H | 1.700±0.073 | 4.28 |  | 84.9 |
|  | NHS I | 1.630±0.114 | 6.99 |  | 81.3 |
|  | NHS J | 1.633±0.019 | 1.16 |  | 81.7 |
|  | NHS G | 1.623±0.058 | 3.56 |  | 81.1 |
| **Mean Recovery±SD (%)** | | | |  | **82.3±1.8** |
| *^1^Samples consisted of ten different normal human sera (NHS)*  *^2^Data was obtained from at least three spiked-sample replicates*  *^3^Coefficient of Variation* |  |  |  |  |  |
